# Sleep Quality and the Mediating Role of Stress Management on Eating by Nursing Personnel

**DOI:** 10.3390/nu11081731

**Published:** 2019-07-26

**Authors:** José Jesús Gázquez Linares, María del Carmen Pérez-Fuentes, María del Mar Molero Jurado, Nieves Fátima Oropesa Ruiz, María del Mar Simón Márquez, Mahia Saracostti

**Affiliations:** 1 Department of Psychology, Universidad Autónoma de Chile, 4780000 Santiago, Chile; 2 Department of Psychology, University of Almería, 04120 Almería, Spain; 3 Department of Psychology, Universidad Politécnica y Artística del Paraguay, 1628 Asunción, Paraguay; 4 Núcleo Científico y Tecnológico en Ciencias Sociales, Universidad de la Frontera, 4811230 Temuco, Chile

**Keywords:** sleep quality, stress management, eating, nursing

## Abstract

(1) Background: The work schedule of nursing personnel often involves double or continuous shifts and sources of stress derived from the work context, making it necessary to ensure their rest and eating habits contribute to a healthy lifestyle. The objective of this study was to analyze the mediating role of stress management on the effect that sleep quality has on uncontrolled and emotional eating by nursing professionals. The Three-Factor Eating Questionnaire-R18 was applied to measure uncontrolled and emotional eating, the Pittsburgh Sleep Quality Index as a measure of sleep quality, and the EQ-i-20M for the stress management component of emotional intelligence. (2) Methods: A sample of 1073 nurses aged 22 to 57 years was selected for this purpose. (3) Results: The main result of this study was that stress management was a mediator in the effect of sleep quality on uncontrolled and emotional eating. Furthermore, low scores for sleeping problems correlated with high scores for stress management. The results also revealed a strong negative association between stress management and uncontrolled and emotional eating. (4) Conclusions: The results are discussed from the perspective of promoting health at work as well as improving the psychosocial wellbeing of nursing professionals and increasing the quality of patient care.

## 1. Introduction

The profile of the nursing professional’s workday, which includes eight- and twelve-hour shifts, working day and night, and sources of intense stress, makes it necessary for them to sleep well to ensure their own health as well as the quality of patient care. In fact, some studies have shown that most nurses and healthcare personnel suffer from poor sleep and about half from moderate-to-excessive somnolescence [1]. These results may be due in part to the rotating and extended shifts which characterize nursing work. It also seems that working long shifts of over twelve hours is associated with burnout, job dissatisfaction, and intention of quitting [2].

Sleeping problems are usually associated with excessive worry, rumination, and significant activation of negative thoughts and emotions. In the clinical and subclinical scope, they are related to mood disorders such as depression and anxiety [3]. In the scope of the organization, they have also been linked with emotional exhaustion [4]. Several studies have shown that sleeping problems correlate positively with both uncontrolled and emotional eating in different populations [5,6]. On the contrary, sleep duration and sleep quality have been associated with healthy diets [7,8]. Good sleep also seems to be linked to highly positive emotions [9], and greater psychosocial wellbeing [10,11].

In addition to the above, the nursing staff are the direct link with patients, taking on their constant care, assistance, and attention. New approaches to health focus service quality on patient-centered care (PCC) [12], where clinical decisions are taken considering the interests and needs of the patients themselves. PCC leads to higher satisfaction, adherence to treatment, and self-management by the patient [13]. Emotional intelligence plays a relevant role in this [14]. Recent studies have also corroborated the fundamental role of emotional intelligence in care professions, and in particular, nursing, showing emotional intelligence to be a predictor of job commitment and a protector against burnout [15,16], with a significant negative correlation between job commitment and burnout in this field [17].

However, today’s world is characterized by change, with various changes occurring one after another in dizzying succession, and at the same time, almost inappreciably, affecting people’s lifestyles. These circumstances have led to shorter sleep–wake cycles and unhealthier eating habits. In this respect, studies have revealed that stress management plays an essential role in these lifestyle habits. Bar-On [18] defined stress management as a dimension of emotional intelligence. It refers to the capacity for self-regulating emotions in stressful situations. This involves the degree of tolerance to stress from frustration (ability to act calmly and perform well under pressure), and control impulses (ability to show low impulsivity and little emotional delay in stressful events). In a study by Cabanach, Souto-Gestal, and Cervantes [19] with health science students, control and acceptance of emotional states facilitated their adaptation to them and decreased their stress response.

To date, the mediating role of psychological variables such as stress management in the relationship between sleep quality and eating habits has largely been unquantified. However, many studies on the relationship of stress management with each of these variables individually have been carried out. Below, some results on the relationship between stress management and eating are discussed, in the knowledge that contextual and cultural factors also influence eating [20,21]. In this respect, the study by Gianini, White, and Masheb [22] demonstrated that problems with emotional regulation could play an important role in emotional eating and obesity. Similar results in the study by Andrei et al. [23] showed that obese individuals had lower scores for emotional intelligence than those with normal weight, in this case, mainly due to not expressing their emotions. With respect to the association between stress management and sleep quality, sleep disturbance has been related to deficient stress management in different countries [24]. Many studies have suggested that mindfulness can contribute to improving emotional regulation and stress management [10,25,26].

**Figure 1:**
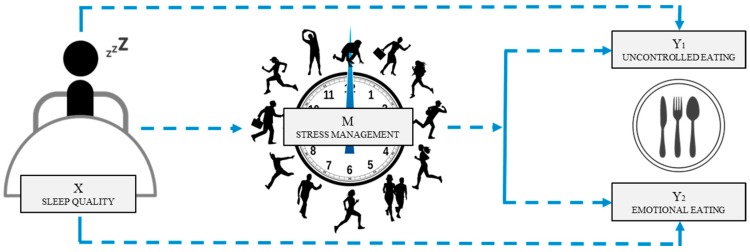
Diagram with the hypothesized relation between the variables.

Considering the findings of the research reported above, the objective of this study was to analyze the mediating role of stress management on the effect that sleep quality has on uncontrolled and emotional eating by nursing professionals. First, it is expected that significant positive associations of sleep and uncontrolled and emotional eating will be found. Second, stress management is expected to correlate negatively with low sleep quality and inappropriate eating (Figure 1).

## 2. Materials and Methods

### 2.1. Participants

From an initial sample of 1094 nurses from Andalusia (Spain), all cases in which random responses were detected or did not complete the entire questionnaire were discarded. After this filtering, the sample consisted of 1073 nurses with an average age of 32.32 years (SD = 6.62, range of 22 to 57 years). By sex, the distribution of the sample was as follows: 14.7% (*n* = 158) men and 85.3% (*n* = 915) women, with an average age of 32.79 (SD = 6.27) and 32.24 years (SD = 6.68), respectively.

### 2.2. Instruments

The Pittsburgh Sleep Quality Index (PSQI) [27], in the Spanish version [28], was used to evaluate sleep. This questionnaire measures sleep quality, distinguishing good sleepers from poor sleepers. Out of a total of 24 items, five are evaluated by a roommate (they do not qualify in the evaluation of the subject being studied), and other remaining elements are self-reported. They evaluate relevant aspects of sleep quality, such as the duration and latency of sleep and the frequency and severity of sleep problems. Based on the subject’s answers, seven components are generated: subjective quality, latency, usual efficiency, disturbance, use of sleeping medication, and daytime dysfunction. A global score on sleep quality is found from the sum of these partial components. In the Spanish version of the instrument, the authors [29] revealed reliability indices that ranged between 0.67 and 0.81, after application in different populations.

In addition, to evaluate the eating variables, a short version of the original TFEQ [30] was applied, specifically the Three-Factor Eating Questionnaire-R18 [31]. In this study, the adaptation of Pérez-Fuentes, Molero, Gázquez and Oropesa [32] was used for the nursing population. The questionnaire consists of 18 elements with four response options, which refer to three dimensions of eating behavior: (a) uncontrolled feeding (loss of control over eating with a subjective feeling of hunger that leads to a tendency towards excessive intake); eat emotionally (under the control of emotional cues, responding to negative emotions with food intake); cognitive restriction (ability to voluntarily restrict food to promote weight loss). The questionnaire shows adequate reliability (0.75–0.87) in the general population [31], and was optimal in nurses (0.85–0.90) [32]. In this case, reliability indexes ranging between 0.74 and 0.89 were obtained for the scales.

Finally, as a measure of emotional intelligence, the Brief Inventory of Emotional Intelligence for Elderly People (EQ-i-20M) was used [33]. It is an adapted version of the Inventory of Emotional Intelligence: Youth Version by Bar-On and Parker [34], validated by the authors for the Spanish adult population. The EQ-i-20M is composed of 20 elements, in which the subject must choose his answer from among four possible options. The response mode is based on a Likert scale. The questionnaire offers scores on five factors of emotional intelligence (intrapersonal, interpersonal, stress management, adaptability, and mood); however, to respond to the objectives proposed in this case, we will focus in the component of Stress Management (α = 0.83).

### 2.3. Procedure

The study was approved by the Bioethics Committee of the University of Almeria, Ref. UALBIO2017/011. Compliance with the ethical standards of the investigation was guaranteed. For this, prior to the implementation of the questionnaires, the participants were informed of the objectives of the study and the anonymity of their responses. Informed consent was required from all participants before their inclusion in the study. The implementation of the questionnaires was carried out via access to a web platform, participation was disseminated through health organizations (health centers, hospitals, etc.), where participants could register their answers online anonymously. The survey was completed during the months of April and May of 2018. Regarding the control of random or incongruent responses, a series of questions designed for this purpose were introduced. Therefore, certain answers to these control questions allowed to detect the null cases, which were discarded from the sample.

### 2.4. Data Analysis

First, bivariate correlations were performed to check the relationships between variables, which would later be included in the computation of the causal analyses. The descriptive statistics of these variables were also presented. The differences in stress management between those with sleeping problems and those without were identified by the Student’s *t*-test for independent samples. The variable was first dichotomized for this following the authors’ proposal [27], where PSQI Global > 5 suggests that the subject would have severe difficulties in at least two areas, or moderate difficulties in more than three areas of sleep. This gave us two groups: “Good sleep quality” (coded as 0 = without sleeping problems), and “Poor sleep quality” (coded as 1 = with sleeping problems).

The SPSS macro by Hayes [35] was used to estimate the simple mediation model. This resource enables regression models to be computed with information on indirect effects, as an alternative resource to overcome the limitations of the classic proposal of Baron and Kenny [36]. Bootstrapping (5000 bootstraps) enabled estimation of 95% confidence intervals and determination of the effect of the mediator variable.

## 3. Results

### 3.1. Descriptive and Correlational Analyses

Classification by the sleep quality variable showed that 60% (*n* = 644) had trouble sleeping (PSQI Global > 5, Poor sleep quality) and 40% (*n* = 429) did not (PSQI Global ≤ 5, Good sleep quality). Table 1 shows the characteristics of the participants, both for all of the sample and for the groups according to the presence or absence of sleep disturbances (poor sleep quality vs. good sleep quality).

The descriptive statistics and analysis of correlations between the variables (PSQI Global, TFEQ-R18 factors, and intelligence emotional dimensions), are presented in Table 2. To measure the quality of sleep, the total PSQI score was taken. The authors [27,28] established a range of 0 to 21 points, where a score of 0 means that there is no sleep problem, while a score of 21 means that there are serious problems in all areas or dimensions of quality of the dream. Therefore, a higher score in the PSQI implies a poor quality of sleep, while low scores indicate a good quality of sleep.

**Figure 2:**
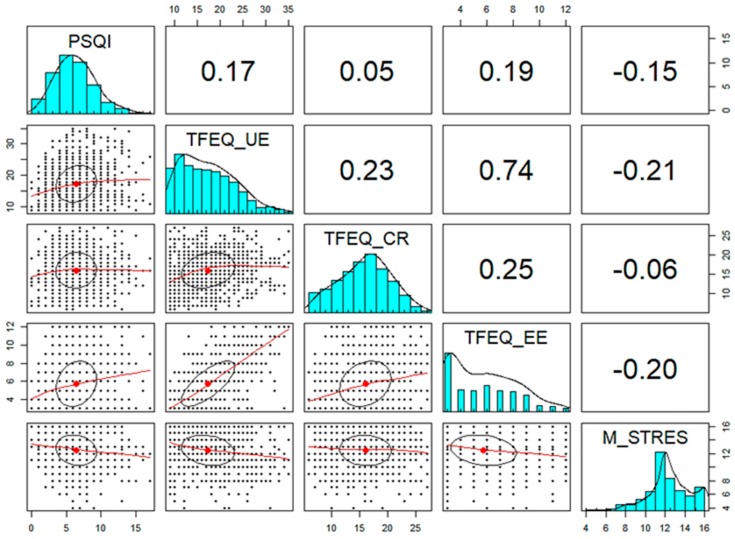
Correlation matrix: sleep quality, stress management, and eating. Note. PSQI = Sleep quality; TEFQ_UE = Uncontrolled eating; TFEQ_EE = Emotional eating; TFEQ_CR = Cognitive restriction; M_Stress = Stress management.

Data were found that confirmed the existence of a positive correlation between the predictor variable (total PSQI score) and two of the variables referring to eating: uncontrolled eating (*r* = 0.17, *p* < 0.001) and emotional eating (*r* = 0.19, *p* < 0.001). No correlation was found between cognitive restriction and sleep quality, or stress management (Figure 2).

Stress management showed negative correlations with sleep quality (*r* = −0.14, *p* < 0.001), with uncontrolled eating (*r* = −0.21, *p* < 0.001), and with emotional eating (*r* = −0.20, *p* < 0.001). Therefore, these are the variables that were later included for testing the mediation models proposed.

In addition to the above, when the sleep quality groups were compared, statistically significant differences were found in Stress management (*t*(1071) = 3.99; *p* < 0.001; *d* = 0.25), where those who had no sleeping problems scored highest (*M* = 12.82; SD = 2.13), compared to those who had problems sleeping (*M* = 12.27; SD = 2.27).

### 3.2. Mediation Models for Estimating the Effects on Uncontrolled and Emotional Eating

The mediation analysis was performed based on the following mediation hypothesis: Having problems sleeping involves less ability to manage stress, which has negative repercussions on controlling eating, as well as on emotional eating.

To compute the models, the total score on the PSQI was taken as the independent or predictor variable. In the first model, the dependent variable was uncontrolled eating, and in the second, emotional eating. Thus, two simple mediation models were computed, taking stress management as the mediator (M) in both cases.

**Figure 3:**
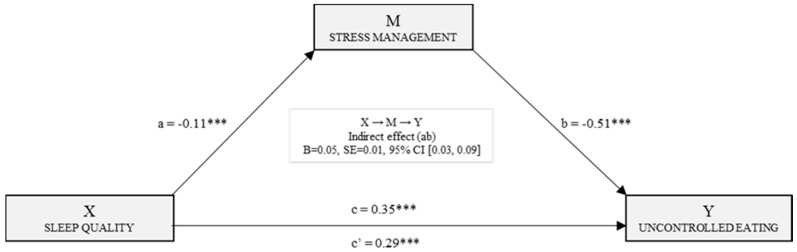
Simple mediation model of stress management in the relationship between sleep quality and uncontrolled eating. Note. *** *p* < 0.001.

Figure 3 shows the direct, indirect, and total effects that result from the simple mediation model for uncontrolled eating. There is a statistically significant effect (B_PSQI_ = −0.11, *p* < 0.001) of sleep quality in stress management. The following regression analysis includes sleep quality and stress management in the model. The effects on the dependent variable (uncontrolled eating) were statistically significant (B_Stress_M_ = −0.51, *p* < 0.001) and (B_PSQI_ = 0.29, *p* < 0.001). On the other hand, after estimating the total effect of the independent variable on the dependent variable, there was a significant effect of sleep quality on the uncontrolled eating (B_PSQI_ = 0.35, *p* < 0.001). Finally, data analyzed by bootstrapping found the indirect effect to be significant [B = 0.05, SE = 0.01, 95% CI (0.03, 0.09)].

**Figure 4:**
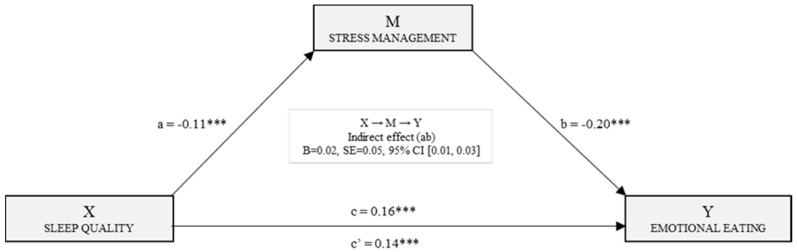
Simple mediation model of stress management in the relationship between sleep quality and emotional eating. Note. *** *p* < 0.001.

Figure 4 shows the simple mediation model for emotional eating. The effects of sleep quality and stress management on emotional eating were estimated based on the second regression analysis. The effects of the independent variable and the mediating variable were significant (B_PSQI_ = 0.14, *p* < 0.001) and (B_Stress_M_ = −0.20, *p* < 0.001). The total effect of sleep quality on emotional eating was also significant (B_PSQI_ = 0.16, *p* < 0.001). Finally, data analyzed by bootstrapping found the indirect effect to be significant [B = 0.02, SE = 0.05, 95% CI (0.01, 0.03)].

## 4. Discussion

The first idea surmised from the analysis of nursing personnel made above, is that the results concerning the mediating role of stress management on the effect of sleep quality on both uncontrolled and emotional eating are conclusive. There are few such studies in the field of healthcare, and in particular, in nursing, so these results are of special interest. Therefore, our main hypothesis, which was, a priori, that we expected to find that the stress management emotional variable would explain the relationship established between the two variables (sleep quality and eating behavior) was confirmed.

Coinciding with previous studies, our data also showed a significant positive relationship between sleeping problems and both uncontrolled (proneness to loss of control of eating) and emotional (inability to resist emotional signals) eating. Therefore, in both cases, poor sleepers also scored higher for this type of inappropriate eating. Previous studies performed on other population samples have also found similar results [5,6]. Specifically, in the work of Blumfield et al. [5], it is suggested that an improvement in the quality of sleep facilitates the processes of weight loss, acting on the ability of the person to control excessive intake. On the other hand, Kilkus et al. [6] report that a low quality of sleep could be associated with certain unbalanced eating patterns in adults. In both studies, the authors hypothesize that the analysis of sleep quality is a valid predictor in relation to the control of intake, beyond the most quantitative measure of sleep (duration of sleep).

In addition, when the association between stress management and sleep quality was analyzed in our study, low scores for sleeping problems correlated with high scores for stress management. Thus, good sleepers showed better stress management and vice versa. These findings support the hypothesis of the relevance of sleep quality to the health of nursing personnel. In the bibliography reviewed, we found similar results, such as those found by Otsuka et al. [24] where sleep disturbance was associated with deficient stress management in samples from different countries. The authors found that lack of rest or poor sleep quality was more present in stressed people and, conversely, the effective use of strategies for stress management was related to an improvement in sleep quality.

Moreover, the results regarding the association between stress management and uncontrolled and emotional eating showed a strong negative association between the two variables. These results provide evidence that high scores in stress management were generally associated with healthier eating behavior. Similar results have been found in other studies [22,23], comparing emotional intelligence in an obese population and another with normal weight, where scores were lower for emotional expression and stress management in obese subjects. Thus, aspects related to emotional management or regulation play an important role in the control of emotional intake [22]. Therefore, it is necessary to pay attention to psychological factors related to emotional management, which can be decisive in interventions related to the problems of eating behavior [23].

Therefore, the role of emotional stress in stress management for coping positively with potentially stressful situations, especially in care professions, and in our case nursing, must be emphasized. The value of mindfulness in reducing stress should also be noted [10,25,26,37].

This study analyzed the cause–effect relationship of sleep quality on eating behavior based on the mediating role of stress management, and found results in favor of stress management in this relationship between variables. However, we must be prudent in generalizing the results, as they are obtained from a cross-sectional study, albeit from of a large sample. In the case of stress management, the authors [33] found that it was the “weakest” component of the scale. However, the sample in that case presented different characteristics to that of the present study (for example, the average age was higher, and they were not active people).

Furthermore, they are obtained from a sample of nurses, a population that is characterized by a greater representation of the feminine sex; therefore, the results must be interpreted in this context. In addition, data were collected from an online questionnaire and could, therefore, be biased, although the social desirability bias is somewhat lower in online surveys.

On the other hand, for future lines of research, it would be advisable to include other variables of interest (lifestyle, caffeine and tobacco consumption habits, work shift, weight status, etc.), which were not contemplated in the present work because it is an analysis of psychological factors, that could be completed with the proposal of an integral model that includes physical, behavioral, or labor performance variables. Another of the limitations in this study is the lack of any consideration of sleep duration, weight gain and its relationship to uninhibited eating behavior, such as that analyzed by Chaput et al. [38]. These authors demonstrated, in a longitudinal study, that a trait of uninhibited eating behavior increased the risk of overeating, with the consequent weight gain, in adults with short duration sleep. This may be analyzed in future studies.

Finally, it should be mentioned, based on these results and coinciding with Dashti et al. [7], that strategies promoting health should emphasize the improvement of sleep quality as an additional health factor, as it impacts stress management, and this in turn, reduces susceptibility to overeating, regulating eating behavior, and weight control.

## 5. Conclusions

The main conclusion of this study is that stress management acts as a mediator in the relationship between sleep quality and uncontrolled and emotional eating. The data found serve as the basis for designing intervention programs for improving sleep quality in nursing personnel, with closer attention paid to stress management, pursuing adequate positive coping mechanisms related to eating behavior, which are relevant for health and individual wellbeing. Therefore, awareness campaigns on the benefits associated with sleep, protocols that favor healthy sleep habits, and reduction and stress management techniques form some of our intervention proposals.

This type of study enables progress in promoting both personal and occupational health, as well as improving the psychosocial wellbeing of nursing professionals (predomination of positive emotions, increase in satisfaction with life and job, improvement of interpersonal relations), and as a consequence, an increase in patient care quality. This means that the organization will be able to count on healthier, more satisfied, and committed workers, increasing their enthusiasm and positive attitude in the performance of their functions, and also, diminish costs associated with job dissatisfaction.

In conclusion, we suggest that some possible lines of future research be directed at corroborating the findings in other population groups (doctors, physiotherapists, etc.) to contribute to the generalization of the results in the care context. The role of positive emotions and stress management in developing personal resources, such as self-esteem, for example, and their relationship with eating behavior and occupational health of healthcare personnel should be studied further.

## Figures and Tables

**Table 1:** Characteristics of the participants. Total sample and subsample attending to sleep difficulties.

	Total Sample (*n* = 1073)	PSQI > 5 (*n* = 644) Poor Sleepers	PSQI ≤ 5 (*n* = 429) Good Sleepers
Sex			
Man	14.7% (*n* = 158)	13.7% (*n* = 88)	16.3% (*n* = 70)
Woman	85.3% (*n* = 915)	86.3% (*n* = 556)	83.7% (*n* = 359)
Civil status			
Single	50.6% (*n* = 543)	48% (*n* = 309)	54.4% (*n* = 234)
Married or stable partner	47% (*n* = 504)	49.2% (*n* = 317)	43.6% (*n* = 187)
Separated or divorced	2.2% (*n* = 24)	2.8% (*n* = 18)	1.4% (*n* = 6)
Widower	0.2% (*n* = 2)	-	0.5% (*n* = 2)
Age	32.32 (SD = 6.62)	32.69 (SD = 6.73)	31.76 (SD = 6.42)
TFEQ-R18			
Uncontrolled eating	17.38 (SD = 5.88)	18.08 (SD = 6.11)	16.32 (SD = 5.36)
Emotional eating	5.75 (SD = 2.50)	6.01 (SD = 2.60)	5.37 (SD = 2.28)
Cognitive restriction	16.08 (SD = 4.55)	16.28 (SD = 4.55)	15.77 (SD = 4.53)
PSQI Global	6.44 (SD = 2.90)	8.27 (SD = 2.16)	3.70 (SD = 1.25)
Perception of health *	3.40 (SD = 0.59)	3.32 (SD = 0.59)	3.52 (SD = 0.57)

Note. * How would you say your state of health is? (Likert scale of 0 = very bad, to 4 = very good).

**Table 2:** Descriptive statistics and correlations between Pittsburgh Sleep Quality Index (PSQI) Global, Three-Factor Eating Questionnaire (TFEQ)-R18 factors, and emotional intelligence variables.

	*M*	*SD*	1	2	3	4	5	6	7	8
1. PSQI Global	6.44	2.90	-							
2. Uncontrolled eating	17.38	5.88	0.17 ***	-						
3. Emotional eating	5.75	2.50	0.19 ***	0.74 ***	-					
4. Cognitive restriction	16.08	4.55	0.05	0.23 ***	0.25 ***	-				
5. Intrapersonal	9.83	2.87	−0.05	−0.01	−0.02	0.02	-			
6. Interpersonal	11.65	2.04	−0.00	0.02	−0.00	0.04	0.37 ***	-		
7. Stress management	12.48	2.23	−0.15 ***	−0.21 ***	−0.20 ***	−0.05	−0.00	0.06 *	-	
8. Adaptability	11.09	2.13	−0.06 *	−0.02	−0.06	0.11 ***	0.40 ***	0.57 ***	0.04	-
9. Mood	11.74	2.33	−0.20 ***	−0.18 ***	−0.22 ***	−0.04	0.36 ***	0.35 ***	0.19 ***	0.54 ***

* *p* < 0.05; *** *p* < 0.001.
